# Comprehensive mass spectrometry lipidomics of human biofluids and ocular tissues

**DOI:** 10.1016/j.jlr.2023.100343

**Published:** 2023-02-10

**Authors:** Glenda Vasku, Caroline Peltier, Zhiguo He, Gilles Thuret, Philippe Gain, Pierre-Henry Gabrielle, Niyazi Acar, Olivier Berdeaux

**Affiliations:** 1 Eye and Nutrition Research Group, Centre des Sciences du Goût et de l'Alimentation, CNRS, INRAE, Institut Agro, Université de Bourgogne Franche-Comté, Dijon, France; 2 ChemoSens Platform, Centre des Sciences du Goût et de l’Alimentation, Institut Agro, CNRS, INRAE, Université Bourgogne Franche-Comté, Dijon, France; 3 INRAE, PROBE Research infrastructure, ChemoSens facility, Dijon, France; 4 Department of Ophthalmology, Biology, Imaging, and Engineering of Corneal Grafts, Faculty of Medicine, Saint Etienne, France; 5 Department of Ophthalmology, University Hospital, Dijon, France

**Keywords:** lipidomic analysis, eye/retina, RPE/Choroid, erythrocytes, plasma, mass spectrometry, age-related macular degeneration, AMD, age-related macular degeneration, Cer, ceramide, chol, cholesterol, DAG, diglyceride, FFA, free fatty acyls, GPh, glycerophospholipids, Hex2Cer, dihexosylceramide, HexCer, hexosylceramide, HILIC, hydrophilic interaction liquid chromatography, HRMS, high-resolution mass spectrometry, IS, internal standard, LPC, lysophosphatidylcholine, LPE, lysophosphatidylethanolamine, PC, phosphatidylcholine, PE, phosphatidylethanolamine, PG, phosphatidylglycerol, PI, phosphatidylinositol, PR, photoreceptor, PS, phosphatidylserine, PUFA, polyunsaturated fatty acids, QC, quality control, RPC, reversed-phase chromatography, RPE, retinal pigment epithelium, SM, sphingomyelin, TAG, triglyceride, VLC, very long chain, VLC-PC, PC species containing VLC-PUFA

## Abstract

Evaluating lipid profiles in human tissues and biofluids is critical in identifying lipid metabolites in dysregulated metabolic pathways. Due to various chemical characteristics, single-run lipid analysis has not yet been documented. Such approach is essential for analyzing pathology-related lipid metabolites. Age-related macular degeneration, the leading cause of vision loss in western countries, is emblematic of this limitation. Several studies have identified alterations in individual lipids but the majority are based on targeted approaches. In this study, we analyzed and identified approximately 500 lipid species in human biofluids (plasma and erythrocytes) and ocular tissues (retina and retinal pigment epithelium) using the complementarity of hydrophilic interaction liquid chromatography (HILIC) and reversed-phase chromatography (RPC), coupled to high-resolution mass spectrometry. For that, lipids were extracted from human eye globes and blood from 10 subjects and lipidomic analysis was carried out through analysis in HILIC and RPC, alternately. Furthermore, we illustrate the advantages and disadvantages of both techniques for lipid characterization. RPC showed greater sensitivity in hydrophobicity-based lipid separation, detecting diglycerides, triglycerides, cholesterol, and cholesteryl esters, whereas no signal of these molecules was obtained in HILIC. However, due to coelution, RPC was less effective in separating polar lipids like phospholipids, which were separated effectively in HILIC in both ionization modes. The complementary nature of these analytical approaches was essential for the detection and identification of lipid classes/subclasses, which can then provide distinct insights into lipid metabolism, a determinant of the pathophysiology of several diseases involving lipids, notably age-related macular degeneration.

Lipids are essential components of cell membranes and have important biological roles, particularly in energy storage, cell signalling, and structure (1, 2). They account for 30% of the human body weight and are found in high quantities in the adipose tissue, where they consist of triglycerides (TAG), 98%, cholesterol (Chol), 0.26%, and glycerophospholipids (GPh), 0.29% (3). Also, the nervous system is rich in lipid content, represented by the brain and peripheral neurons, which conversely are mainly composed of sphingolipids, GPh and Chol in almost equal ratios (4). Within the nervous system, lipids have a different distribution that is structure- and/or cell-specific (5). The retina, which is an extension of the nervous system, is a thin sensory membrane which covers the back of the eye, being responsible for vision (6, 7). The lipid composition of the retina is mainly represented by GPh, particularly glycerophosphoethanolamine and glycerophosphocholine (8). Other lipid classes, such as cholesteryl esters (CE) (9), Chol, and PUFAs, particularly docosahexaenoic acid (10), are also abundant in the retina. Indeed, PUFAs are extensively incorporated into GPh units and play physical roles having an impact on visual transduction (11). Lipids are not only fundamental building blocks for cell membranes but also crucial for cell signalling. Hence sphingolipids and glycosphingolipids are involved in visual signaling and neuronal growth, while protecting against retinal damage (12).

Retinal lipid homeostasis can be disturbed due to genetic, age-related, and environmental factors (13, 14, 15, 16, 17, 18). Recent studies have shown that individuals with high total plasma Chol and TAGs, elevated HDL-Chol, LDL-Chol, and VLDL-Chol, have a significantly higher risk of developing retinal degenerative diseases (19, 20). In the retina, the transport of these lipids between the blood compartment and the photoreceptors (PRs) is mediated by retinal pigment epithelium (RPE) cells. In situations where the lipid transit is disturbed, conspicuous lipid-containing deposits, called “drusen”, accumulate at the basement of RPE cells. This process triggers an inflammatory cascade (21) leading to RPE and PR degeneration as observed in age-related macular degeneration (AMD), the leading cause of visual loss in western countries (22). Lipid metabolism is severely impaired in AMD, resulting in metabolites that appear to be hallmarks of the disease. It was shown that lipid composition of the retina is strongly related to food intake. For instance, linoleic acid, an essential FA, was found to be the most prominent FA in RPE cells (9). The retinal lipid composition is also partly distinguishable due to the presence of very long chain polyunsaturated fatty acids (VLC-PUFAs), which are mainly incorporated into ethanolamine and choline GPh units (23). Their role is not yet fully understood but a retinal deficiency in VLC-PUFAs is associated with the accumulation of drusen material and further PR cell death. On the other hand, studies have shown that genetic mutation in the VLC-PUFA elongase (*ElovL4*) is associated with Stargardt disease type-3, a juvenile autosomal dominant form of macular degeneration (24). Other studies have indicated that certain lipid metabolites, such as sphingosine 1-phosphate (S1P), ceramide 1-phosphate (C1P) and ceramides (Cer), have an important impact on retinal and RPE cells physiology. Thus, C1P stimulates photoreceptor survival and induces PR differentiation in the retina, while Cer emerged as a key mediator of cell death and impairment of the autophagic flux (25). According to a study, it was shown that low concentrations of Cer prevented photoreceptor degeneration, while high concentrations resulted in photoreceptor loss (26). In addition, in a recent metabolomic study on human plasma, Lains and collaborators found that 28 lipid metabolites from the GPh, sphingolipid, and lyso GPh pathways, differed significantly between AMD subjects and controls (27). In the neural retina and RPE, lipids act through different signalling pathways in which different lipid classes and mechanisms are implicated (28). To better understand these mechanisms which are implicated in AMD pathophysiology, it is important to study the entire lipidome by considering all lipid classes/subclasses.

Therefore, this study aims to provide a comprehensive lipidomic approach through the complementarity of utilizing two chromatographic methods [hydrophilic interaction liquid chromatography (HILIC) and reversed-phase chromatography (RPC] coupled to high-resolution mass spectrometry (HRMS), focusing on their specific benefits and drawbacks for screening different classes/subclasses of lipids. In addition, this dual approach can be advantageous for annotating and identifying various lipid classes/subclasses, resulting in a more comprehensive lipidomic profile of these biological samples. The comparison of these analytical methods and the lipidomic data generated on samples from healthy human subjects may be utilized in future research aimed at elucidating the different distribution of these lipid molecules, which appear to have a significant role in the pathogenesis of AMD.

## MATERIALS AND METHODS

### Ethics statement

Collection of the samples from human subjects was conducted in accordance with the guidelines of the Declaration of Helsinki, as previously described (10). A written consent was obtained and the protocol was approved by the local ethics committee (CPP Sud Est I, CHU Saint Etienne, Saint Etienne, France).

### Reagents

Optima LC-MS grade water, methanol, chloroform, acetonitrile, isopropanol, formic acid, ammonium acetate were purchased from Fisher Chemical (Thermo Fisher Scientific, Illkirch, FR). All internal standards (IS) used in this study were from Avanti Polar Lipids, Inc (Alabaster, AL).

### Human tissues

Human biological tissues (retina and RPE/Choroid) and biofluids (erythrocytes and plasma) were obtained from ten human donors for this study. Following death, the bodies were initially stored at 4°C, and the postmortem delay between death and tissue isolation/freezing was less than 24 h. The blood sample was collected in heparinized tubes via venipuncture. Erythrocytes were separated from plasma by centrifuging at 3000 rpm for 10 min at 4°C. The posterior pole of the eye globe was placed on a table with backlighting and the retina was examined under an operating microscope to help determine healthy subjects included in the study. There were no signs of large drusen, severe pigment epithelial alterations, macular haemorrhage, or other chorioretinal pathologies. The vitreous body was carefully removed and the entire neural retina (n = 10) was carefully separated from the RPE/choroid (n = 10). All samples were kept at −80°C until lipid extraction.

### Lipid extraction

Samples from ten human donors (erythrocytes, plasma, retina, and RPE/Choroid) were spiked with 10 μl of a mix of ISs at different concentrations. The lipid species concentrations in the mix of IS were as follows: phosphatidylglycerol *(PG) (14:0/14:0)* at 250 μg/ml, phosphatidylethanolamine (*PE) (14:0/14:0)* at 100 μg/ml, phosphatidylcholine (*PC) (14:0/14:0)* at 100 μg/ml, *PS (14:0/14:0)* at 250 μg/ml, lysophosphatidylethanolamine *(LPE) (14:0)* at 100 μg/ml, lysophosphatidylcholine (*LPC) (14:0)* at 100 μg/ml, *DG (12:0/12:0)* at 250 μg/ml, *TAG d5 (19:0/12:0/19:0)* at 250 μg/ml, *SM (d18:1/12:0)* at 250 μg/ml, *LacCer (d18:1/12:0)* at 250 μg/ml, *GlcCer (d18:1/12:0)* at 250 μg/ml, *Cer (d18:1/12:0)* at 250 μg/ml, *FA (17:0)* at 250 μg/ml, and *CE (17:0)* at 250 μg/ml. Lipids were isolated from tissues (retina, RPE/Choroid) using the Folch's procedure, whereas plasma and erythrocytes were extracted using the Moilanen and Nikkari method (29, 30). The lipid-containing phase was placed in vials and dried under a nitrogen stream. Samples were reconstituted in 200 μl of chloroform/methanol (1:1), vortexed before injection into the LC-MS, and then stored at −30°C after analysis. Quality controls (QCs) were prepared by pooling 10 μl of each sample and injecting them every ten samples throughout the sequence. A system blank composed of methanol and an extraction blank were prepared. The injection order of blanks was every ten samples before the QCs. Lipid analysis was carried out on an LC-MS high-resolution mass spectrometer in both normal and reversed-phase separation conditions, injected alternately.

### LC

#### RPC

Samples were injected in a reversed-phase ACQUITY ultra performance liquid chromatography (UPLC) ethylene-bridged C18 column, 1.7 μm, 2.1 mm × 100 mm (Waters). Mobile phase A consisted of acetonitrile/water 50:50 (v/v) and phase B consisted of acetonitrile/isopropanol/water 10:88:2 (v/v) with 10 mM ammonium formate and 0.1% formic acid for both phases. The solvent-gradient system comprised as follows: 0 min A/B (%) 60/40, 10–12 min A/B (%) 0/100, and 12.1–17 min A/B (%) 60/40. The flow rate was 400 μl.min^−1^ and the column temperature were set at 50°C.

#### HILIC

Separation of samples was performed on an ACQUITY UPLC ethylene-bridged HILIC column, 1.7 μm, 2.1 mm × 100 mm (Waters). Mobile phase A consisted of acetonitrile/water 96:4 (v/v) and phase B consisted of acetonitrile/water 50:50 (v/v). For both phases, ammonium acetate (10 mM) was added. The solvent-gradient system comprised as follows: 0–0.33 min A/B (%) 95/5, 0.33–5.33 min A/B (%) 80/20, 5.33–6.67 min A/B (%) 60/40, and 6.67–10 min A/B (%) 100/0. The flow rate was chosen at 1200 μl.min^−1^ and the column temperature were set at 50°C.

#### MS

Acquisition of mass spectra was performed on a high-resolution mass spectrometer Orbitrap Fusion™ Tribrid™ (Thermo Fisher Scientific, San Jose, CA). Lipid extracts were separated by LC and the column effluent was directly introduced into the EASY-Max Next Generation Atmospheric Pressure Ionization source (Heated-ESI mode), (Thermo Fisher Scientific, San Jose, CA). The acquisition was performed in positive and negative runs, separately, in MS and MS^2^. The method summary for the full MS scan experiment was as follows: the infusion mode was LC with an expected LC peak width at 30 (s), at a charge state of one with an internal mass calibration EASY-IC™. The ion source type used was H-ESI with a static spray voltage at 3500 V and 2800 V in positive and negative ionization mode, respectively. Sheath, auxiliary, and sweep gas were maintained at 60, 20, and 1 respectively, in arbitrary units. The ion transfer tube temperature was 285°C and the vaporizer temperature was set at 370°C. The detector was an Orbitrap with a resolution of 120.000 on a *m/z*range of 200–1600, using quadrupole isolation. The RF lens (%) was 50, while the normalized automatic gain control target (%) was maintained at 112.5. The maximum injection time was 100 (ms) and the data type was acquired in centroid. The method for the MS^2^ negative ion mode experiment was performed using filters such as dynamic exclusion with a duration of 15 (s) and mass tolerance of 5 ppm. For the MS^2^ a data-dependent mode was chosen with a scan in ddMS^2^ orbitrap high collision energy. The isolation mode was performed on a quadrupole with an isolation window *m/z* at 1.2. The collision energy was fixed at 27 eV and the data type was acquired in centroid.

#### Data analysis and processing

We optimized the previous laboratory chromatographic separation method from HPLC to ultra performance liquid chromatography, and therefore the total run time for HILIC (31) and RPC (32) was reduced from 45 to 10 and 17 min, respectively. QCs were analyzed in triplicate, initially in MS^2^ scan in negative and positive ionization mode, to allow for lipid species annotation, and then in MS full scan, while samples were only analyzed in MS full scan in positive and negative ionization mode, separately. Raw files were converted to a readable format, *mzXML,* and then processed in R, including peak extraction and normalization, resulting in the generation of specific files containing data on *m/z*, ppm, and peak intensities. Raw files were visualized with *Xcalibur 4.2* (https://www.thermofisher.com/order/catalog/product/OPTON-30965) and *Freestyle 1.8* (*https://assets.thermofisher.com/TFS-Assets/CMD/manuals/man-xcali-97962-freestyle-14-user-manxcali97962-en.pdf*), whereas *LipidSearch 4.1* (https://www.thermofisher.com/fr/fr/home/industrial/mass-spectrometry/liquid-chromatography-mass-spectrometry-lc-ms/lc-ms-software/multi-omics-data-analysis/lipid-search-software.html) was used for lipid annotation. Ions were identified based on respective adducts (H^−^, OAc^−^, H^+^, NH_4_^+^). Results were compared to the manual annotation of all parent ions from MS^2^ spectrum elucidation. Finally, R Studio was used to acquire and process all data. Raw MS files were converted to *mzXML* format using *MSConvertGUI 64 bit* (https://proteowizard.sourceforge.io/download.html) and then processed using a preestablished database of parent molecular ions based on their exact *m/z* mass for each lipid species. Prior annotation via *LipidSearch* and MS^2^ spectrum elucidation of all lipid species in the QC samples was used to establish the database of lipid classes and species. Minimum and maximum retention time windows were set manually for each lipid class, while the *m/z* tolerance was chosen at 10 ppm.

## RESULTS

### Human donors’ characteristics

Ten subjects participated in the study, three females and seven males, all caucasians. The median age of donors was 87.50 years (mean SD: 87.4 ±6.55 years). Erythrocytes, plasma, retina, and RPE/choroid were collected from each subject. The median postmortem delay was 12.5 h (mean SD: 14.2±7.02 h), with a minimum and maximum of 3 h and 24 h, respectively (Table 1).

**Table 1:** Human’s donor characteristics

Human donor's Characteristics				
Subject	Gender	Age	Postmortem Delay(h)	Collected Tissues
1	M	87	3	erythrocytes, plasma, retina, RPE/Choroid
2	F	93	8	erythrocytes, plasma, retina, RPE/Choroid
3	M	86	13	erythrocytes, plasma, retina, RPE/Choroid
4	M	93	24	erythrocytes, plasma, retina, RPE/Choroid
5	M	97	10	erythrocytes, plasma, retina, RPE/Choroid
6	M	92	12	erythrocytes, plasma, retina, RPE/Choroid
7	M	79	19	erythrocytes, plasma, retina, RPE/Choroid
8	M	82	10	erythrocytes, plasma, retina, RPE/Choroid
9	F	77	19	erythrocytes, plasma, retina, RPE/Choroid
10	F	88	24	erythrocytes, plasma, retina, RPE/Choroid
Mean		87.4	14.2	
SD		6.552	7.021	
Median		87.5	12.5	
Interquartile range		9.750	9	
Minimum		77	3	
Maximum		97	24	
Range		20	21	

### General workflow

**Fig. 1:**
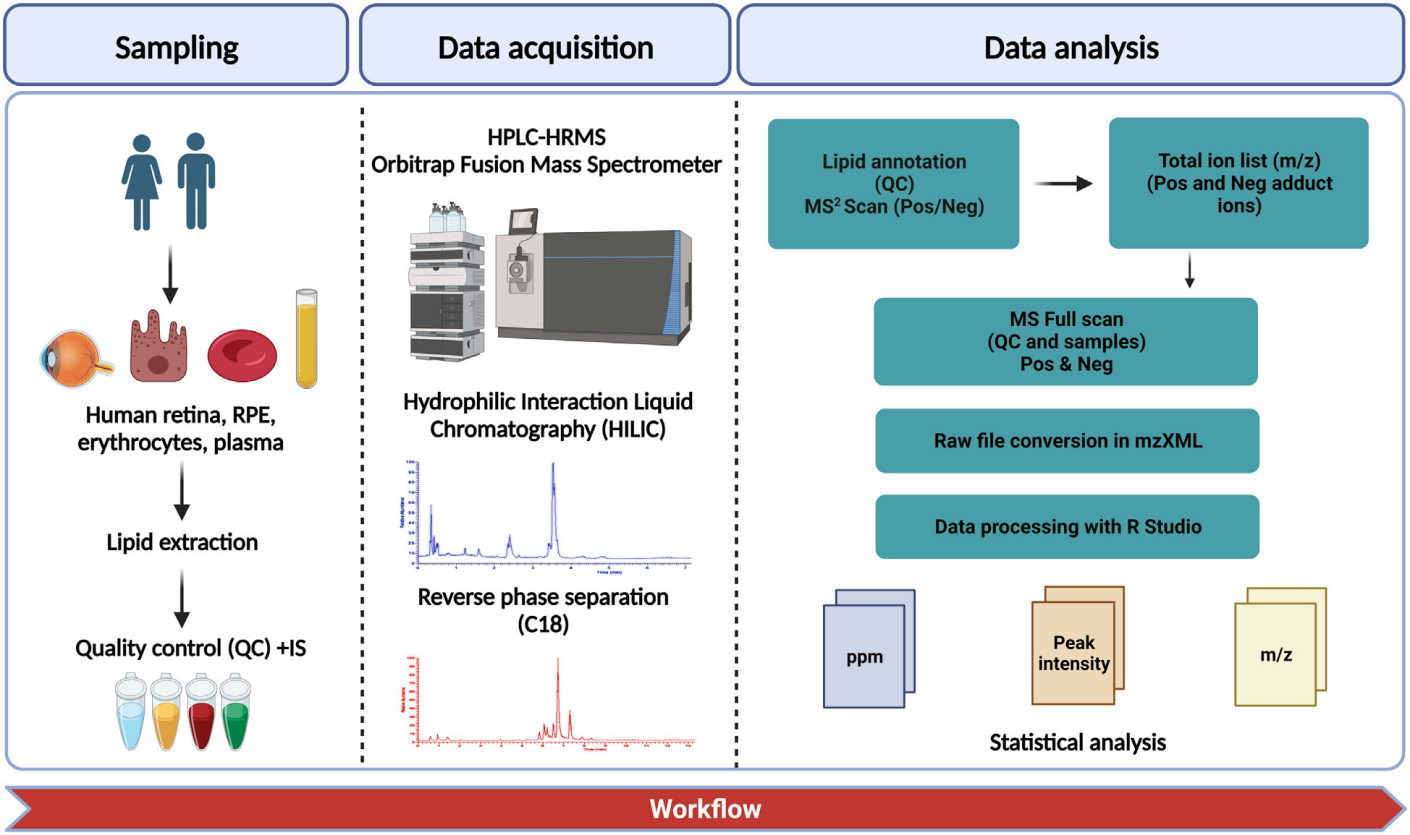
General workflow of the study. Human retina, retinal pigment epithelium (RPE/Choroid), erythrocytes and plasma were analyzed as lipid extracts through Hydrophilic Interaction Liquid Chromatography (HILIC) and reversed-phase chromatography (RPC), coupled to high-resolution mass spectrometry. Data analysis was performed through R and ion’s peak intensities were extracted from conversed files in *mzXML*. Files were matched with the integrated lipid database that was previously prepared and *m/z* masses were extracted with their respective intensities. *Created with:* BioRender.

The overall design of the study was depicted in Fig. 1. Erythrocytes, plasma, retina, and RPE/Choroid lipid samples were extracted and the lipid extract was separated through two alternating chromatographic techniques (HILIC and RPC) in positive and negative ionization mode. After separation, lipid extracts were introduced to the ESI ionization source connected to Orbitrap Fusion HRMS for MS and MS^2^ scan. After data collection, lipid annotation was accomplished using LipidSearch and manual spectral elucidation of MS^2^ scan files, whereas relative lipid quantification was attained using MS full scan files. R 4.0.2 was used to process data, resulting in the generation of files containing information on ppm, peak intensity, and *m/z*. The annotation of each lipid species within each sample, as well as the relative distribution, exact mass, and ppm features of each lipid species were reported.

## METHOD VALIDATION

Lipid annotation was achieved from MS^2^ scans in positive and negative ionization mode in HILIC and RPC. Manual annotation on precursor ions confirmed the lipid composition of species after using *LipidSearch* as a database for lipid identification. Such approach resulted in the annotation of 15 lipid classes and approximately 500 lipid species. Each precursor ion’s relative intensity was extracted automatically via R and the results were computed in a centroided mode. Each *mzXML* file was compared to the database of precursor ions, which was prior established, and then three separate files *(.csv)* were generated with data on *m/z*, ppm, and peak intensities. The lipid database comprising of *m/z* and all the lipid species annotated through HILIC (supplemental Table S1) and RPC (supplemental Table S2), is available in the supplementary section. To validate this methodology, results of the automatic processing of peak extraction from R, were compared with those obtained by manually extracting peak intensities from all QC samples through *Freestyle* software. We demonstrate a positive correlation between automatic peak extraction through R and manual extraction directly from *Freestyle* software for the two techniques (RPC and HILIC), in positive and negative ionization mode. (supplemental Fig. S3).

### Relative distribution of lipid classes

**Fig. 2:**
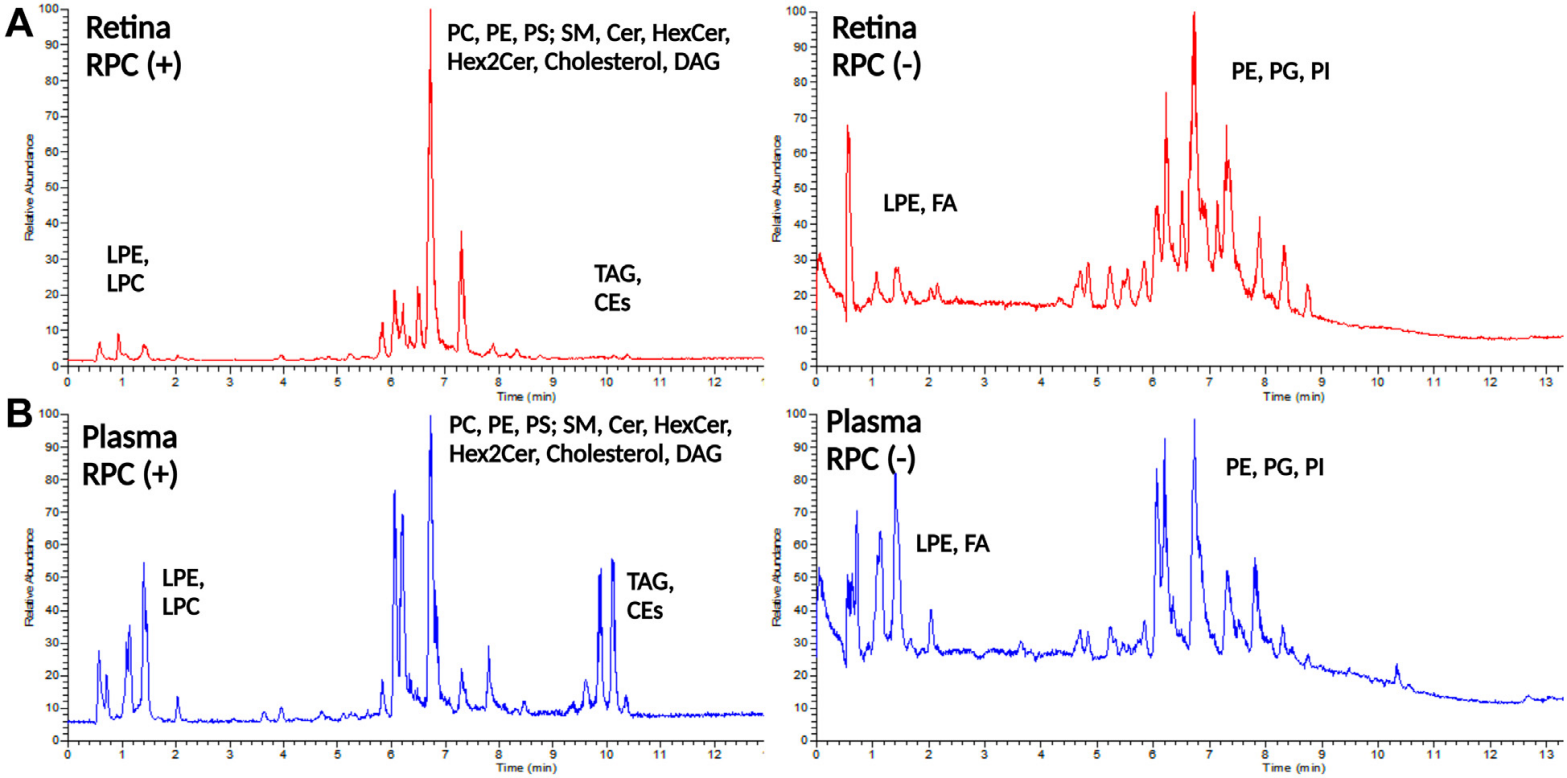
A: Total ion chromatogram (TIC) of QC samples of retina (red) and (B) TIC of plasma (blue), in positive and negative ionization mode in RPC. RPC, reversed-phase chromatography; QC, quality control.

**Fig. 3:**
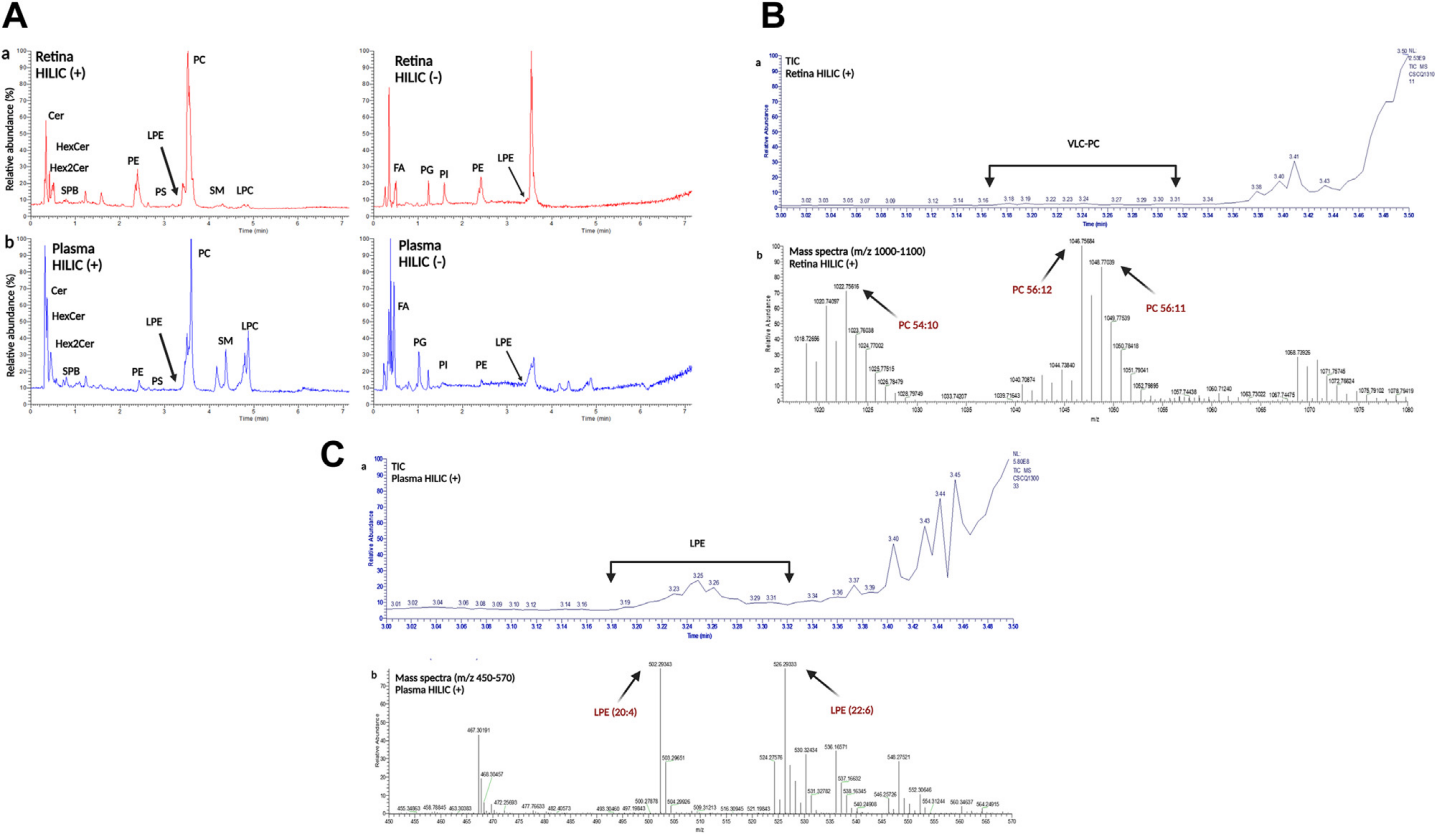
A: (a), Total ion chromatogram (TIC) of QC samples of retina (red) and (b) TIC of plasma (blue), in positive and negative ionization mode in HILIC. B: (a): Extracted total ion chromatogram (TIC) (RT:3-3.5 minutes), showing the elution window for VLC-PC in the retina, in HILIC in positive ionization mode. (b): Mass spectra extracted from the RT:3–3.5 minutes, at *m/z* 1000-1100, representing a few of the VLC-PC species that are present in the retina. C: (a) Extracted total ion chromatogram (TIC) (RT:3–-3.5 minutes), showing the elution window for LPE in plasma, in HILIC in positive ionization mode. (b): Mass spectra extracted from the RT:3–-3.5 minutes, at *m/z* 450–-570, representing a few of the LPE species. HILIC, hydrophilic interaction liquid chromatography; LPE, lysophosphatidylethanolamine; VLC-PC, PC species containing VLC-PUFA; QC, quality control.

**Fig. 4:**
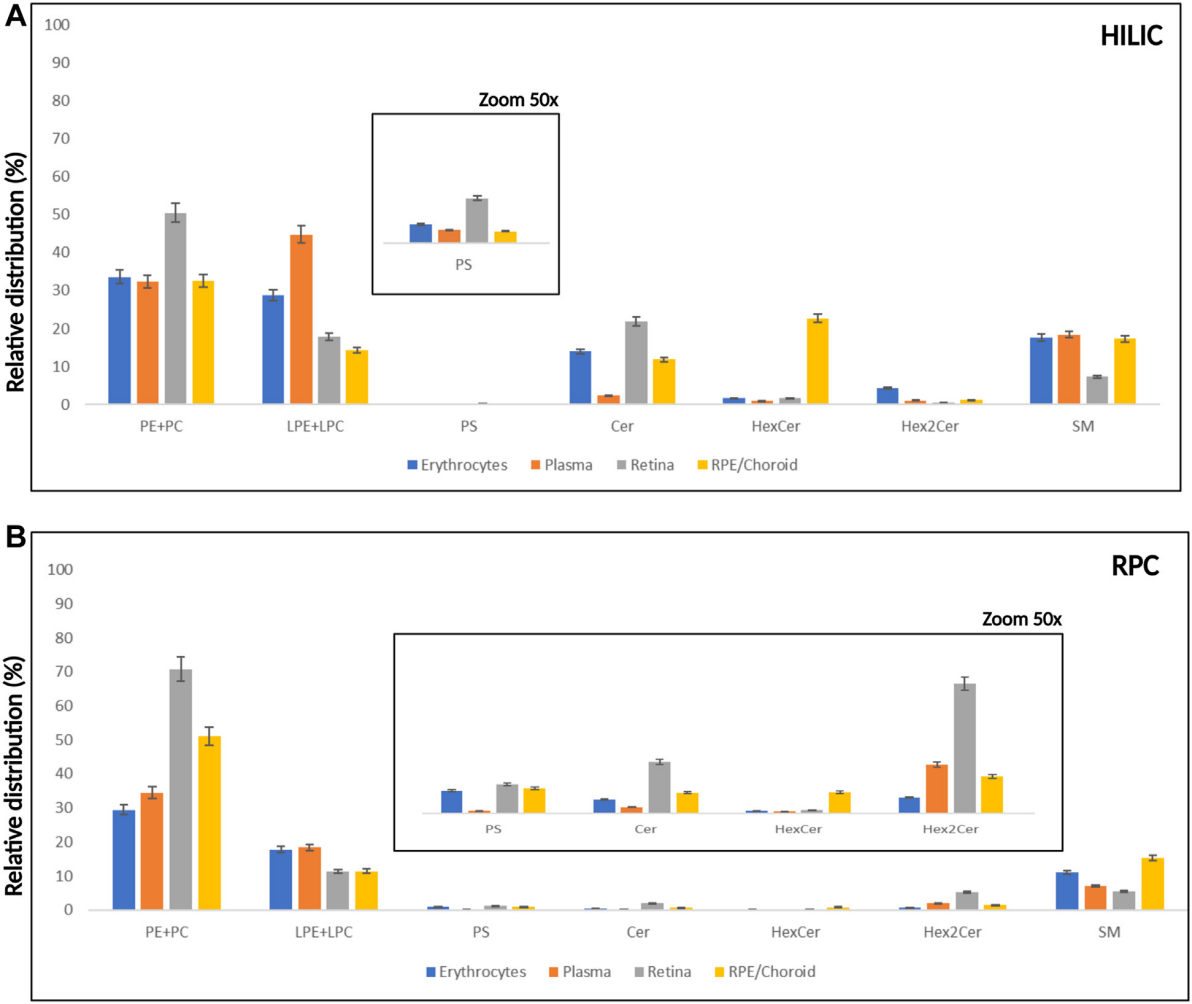
A: Relative lipid distribution of lipid classes in (%), PE+PC (mean SD: 37.24± 6.59), LPE+LPC (mean SD: 26.42± 10.34), PS (mean SD: 0.12± 0.06), Cer (mean SD: 12.53± 5.38), HexCer (mean SD: 6.75±7.95), Hex2Cer (mean SD: 1.78± 1.23), and SM (mean SD: 15.17± 3.91) in RPC in positive ionization mode, evaluated in erythrocytes, plasma, retina, and RPE/Choroid. B: Relative lipid distribution of lipid classes PE+PC (mean SD: 46.48± 14.51), LPE+LPC (mean SD: 14.78± 3.36), PS (mean SD: 0.79± 0.34), Cer (mean SD: 0.92± 0.56), HexCer (mean SD: 0.29±0.27), Hex2Cer (mean SD: 2.32± 1.44), and SM (mean SD: 9.77± 3.44) in HILIC in positive ionization mode, evaluated in erythrocytes, plasma, retina, and RPE/Choroid. Lipid classes such as PS, Cer, HexCer, Hex2Cer were evidenced (zoom 50x) in HILIC and RPC. Cer, ceramide; HexCer, hexosylceramide; Hex2Cer, dihexosylceramide; HILIC, hydrophilic interaction liquid chromatography; LPE, lysophosphatidylethanolamine; LPC, lysophosphatidylcholine; PC, phosphatidylcholine; PE, phosphatidylethanolamine; PS, phosphatidylserine; RPC, reversed-phase chromatography; RPE, retinal pigment epithelium; SM, sphingomyelin.

The lipid profile of erythrocytes, plasma, retina, and RPE/Choroid was determined through RPC (Fig. 2). and HILIC (Fig. 3). Cer, dihexosylceramide (Hex2Cer), hexosylceramide (HexCer), PE, PC, phosphatidylserine (PS), LPC, LPE, sphingosine bases, and sphingomyelin (SM) were detected as protonated adducts [M+H]^+^. Lipid classes such as PE, LPE, phosphatidylglycerol (PG), phosphatidylinositol (PI), and free nonesterified fatty acyls (FFA) were detected as deprotonated [M-H]^−^ adducts in negative mode. CE, TAG, followed by Chol and diglycerides (DAG), were detected only in RPC, as ammonium adducts [M+NH_4_]^+^ in positive ionization mode (Fig. 2), as we report no signal from these molecules in HILIC (Fig. 3A). Such response might be explained due to the column properties and the chemical characteristics of the molecules which contribute to the overall molecular hydrophobicity. The hydrophobicity of a molecule is determined by the length of the carbon chain and the presence of unsaturations (double bonds in this case); thus, longer carbon chains with multiple unsaturations have higher hydrophobic properties. These molecules are therefore more retained in the RPC column than polar compounds, which are less retained and consecutively elute faster. It should be noted that depending on the type of samples being analyzed and the lipids of interest, this can be significant. Chol, CE, and TAG, for example, are abundant in plasma/serum, so RPC can be the method of choice for their analysis. HILIC was chosen as a secondary method to evaluate the lipid profile of erythrocytes, plasma, retina, and RPE/Choroid. In contrast to RPC, lipids in HILIC were primarily separated based on their polarity. Furthermore, PC species containing VLC-PUFA (VLC-PC) were detected via HILIC and RPC, only in the retina, as this subclass is only found in retinal tissues (Fig. 3B) (23). Furthermore, VLC-PC eluted in similar RT with another class, such as LPE, that is also detected in positive ionization mode. Additionally, in Fig. 3C we show that the extracted chromatographic peak of plasma, along with the extracted ions appear to belong to the LPE and not VLC-PC. Indeed, no molecular ions corresponding to VLC-PC appear in the selected mass range spectra. Identification of these molecular ions was done through spectral elucidation through MS^2^ fragmentation. Figure 4A, B depicts the relative intensity of all common lipid classes detected in both HILC and RPC. Based on their relative intensity, we can conclude that for the same lipid subclass, chromatographic separation produces a different response, which is then translated into a varying intensity for that specific lipid class. Moreover, depending on the chromatographic affinity and conditions used to separate these classes, each lipid class or species has a different behavior. The general distribution of lipid’s intensity in both modes in erythrocytes, plasma, retina, and RPE/Choroid is given for each class, as a (%) relative intensity distribution. In RPC and HILIC, the intensity of PC and PE in positive was presented as their total sum, as well as that of LPE and LPC. This was done to present a general and comparable intensity in RPC and HILIC, between these classes only in positive, while considering the presence of some isobaric species between PE and PC and between LPE and LPC. The separation of these isobaric species cannot be achieved in RPC in positive ionization mode, because these species have very similar RT and exact mass. For instance, due to their exact masses, PE 36:1, *m/z* 746.5694 and PC 33:1, *m/z* 746.5694 cannot be distinguished through RPC. Furthermore, differentiation between these classes in RPC is difficult with our method because coelution occurs within the same retention time (RT: 4.5–9 min). However, due to their different exact masses, we can easily distinguish these species in negative ionization mode. Furthermore, in HILIC, these species can be distinguished in positive mode based on different RT windows. In HILIC, the PE subclass eluted at (RT: 2–2.7 min), whereas the PC subclass eluted at (RT: 3–4 min). Other isobaric lipid species from the PE and PC subclasses were evidenced (supplemental Table S4). In positive, the same result was observed for LPE and LPC, where their relative distribution was presented as a sum of both species compared to the overall intensity, in order to be easily compared between RPC and HILIC, considering the presence of isobaric species between these subclasses. (Fig. 4). For example, LPE (22:1), at *m/z* 536.3711 and LPC (19:1) at *m/z* 536.3711 cannot be distinguished through RPC in positive in ionization mode due to exact mass and similar RT time window. However, these species can be distinguished in negative ionization mode, based on different masses. Furthermore, we can distinct them in HILIC mode, as in this case the RT time of these species will differentiate them. For these subclasses the RT in HILIC were as follows: LPE the (RT: 2.9–3.5 min) and for LPC the (RT: 4.6–5 min) (supplemental Table S5). PS, Cer, HexCer, Hex2Cer, and SM were also found to be common lipid subclasses between HILIC and RPC. Overall, the distribution of lipids between these classes is comparable but there is a significant difference in intensity. The relative distribution of PS, Cer, HexCer, and SM in HILIC and RPC is comparable and this tendency is better visualized after zooming in the graphic bars. However, Hex2Cers’ intensity was shown to be overestimated in RPC compared to HILIC.

**Fig. 5:**
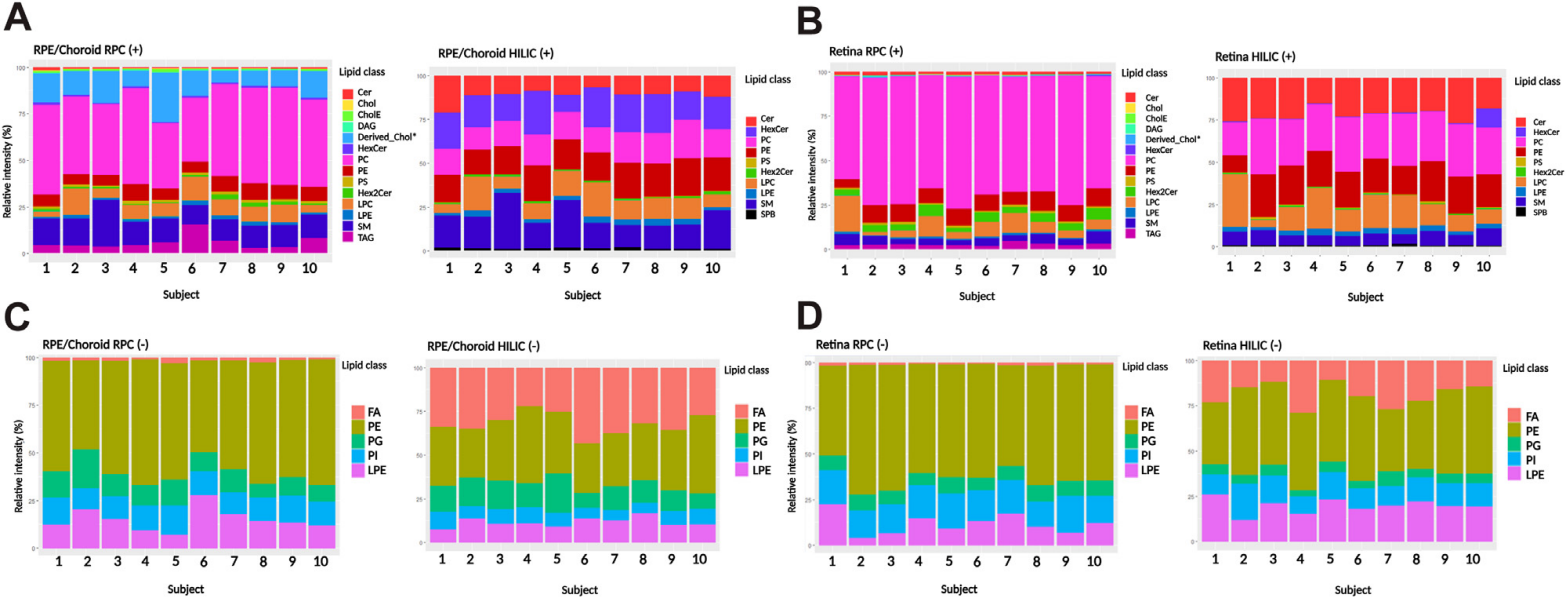
A: Relative lipid distribution of ten subjects’ lipid profile in positive ionization mode in RPC and HILIC in RPE/Choroid. B: Relative lipid distribution of ten subjects’ lipid profile in positive ionization mode in RPC and HILIC in retina. C: Relative lipid distribution of ten subjects’ lipid profile in negative ionization mode in RPC and HILIC in RPE/Choroid. D: Relative lipid distribution of ten subjects’ lipid profile in negative ionization mode in RPC and HILIC in retina. HILIC, hydrophilic interaction liquid chromatography; RPC, reversed-phase chromatography; RPE, retinal pigment epithelium.

Furthermore, we investigated the relative intensity of lipid classes for each chromatographic method separately which is evidenced in Fig. 5A, B in positive ionization mode and in Fig. 5C, D, in negative ionization mode, for RPE/Choroid and retina. The intensity of each lipid class is compared to the overall intensity of all lipid classes analyzed separately through RPC and HILIC method. The relative distribution of lipid classes in RPC and HILIC in retina and RPE/Choroid in positive mode was presented from ten subjects (Fig. 5A, B), while the relative distribution of PE, PI, PG, LPE and FFA was evaluated in negative mode (Fig. 5C, D). We highlight that in RPC graphic representation, the intensity of PC might be overestimated due to the presence of coeluting isomeric peaks between PC and PE. A difference in relative lipid’s distribution was visualized for *subject 10*, which presented a higher value of HexCer class in the retina (Fig. 5B), compared to other subjects. Additionally, in the RPE/Choroid *subject 6* presented a higher relative intensity of LPC class in positive ionization mode (Fig. 5A) and a higher intensity for the FFA class in negative ionization mode in HILIC (Fig. 5C).

### Comparative assessment of RPC and HILIC

RPC and HILIC conditions were chosen for the analysis of lipid classes in lipid extracts of erythrocytes, plasma, retina and RPE/Choroid, as complementary techniques. Both techniques were tested on different lipid matrices, that differ at some extent on their lipid composition. Most of the classes were visible in both RPC and HILIC (PE, PC, PI, PG, PS, LPE, LPC, FA, Cer, HexCer, Hex2Cer, SM), and other classes were only visible through RPC (Chol, CE, DAG, and TAG).

#### Neutral lipids (CE and TAG), Chol, and DAG are efficiently detectable through RPC

Neutral lipids such as CE and TAG which lack charged groups in order to be detected need to be ionized. In the UPLC-ESI-MS^2^ and MS full scan analysis of Chol, CE, DAG, and TAG, they were mainly detected as ammonium adducts [M+NH_4_]^+^. TAG and CE are highly hydrophobic structures and they are more retained by the RPC column. Consequently, in this method they coeluted at a RT range of 9–11 min, in positive ionization mode (Fig. 2A, B). Meanwhile, DAG coeluted before TAG. Such behavior might be explained by its overall less hydrophobic structure compared to TAG, due to the presence of two fatty acyl chains instead of three in TAG. Indeed, DAG coeluted with other lipid classes such as GPh, at a (RT: 5–9 min) (Fig. 2A, B). CE were detectable in RPC in positive mode, as ammonium adducts, but there was a noticeable loss of cholesterol moiety from the CE structure, giving a specific fragment at *m/z* 369.35 corresponding to a dehydrated cholesterol [M+H-H_2_O]^+^ (33). Due to suppressive ionization processes cholesterol was fragmented from the CE structure as a dehydrated moiety at an (RT: 9–11.5 min). In the text and figures, the fragmented Chol from CE was denominated as “derived cholesterol”.

### PC, PE, PS, and lyso GPhs (LPE, LPC) are efficiently separated through HILIC

**Fig. 6:**
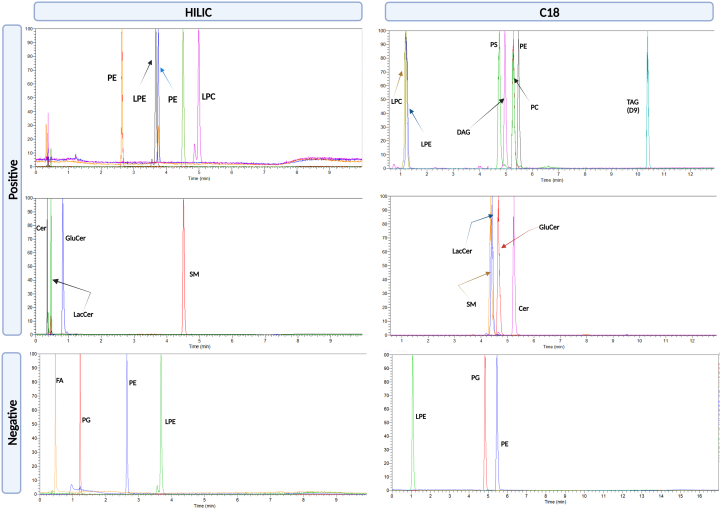
Lipid analysis of a mixture of internal standards of 13 lipid classes, separately analyzed in both ionization modes, in RPC and HILIC. HILIC, hydrophilic interaction liquid chromatography; RPC, reversed-phase chromatography.

PC and PE were both detectable in RPC, with PC being detected specifically in positive mode and PE in both ionization modes. It must be noted that PC and PE species might be confounded due to isomeric species sharing exact *m/z*, which cannot be differentiated through our method in positive mode in RPC. In contrast, PE and PC are easily distinguished in HILIC positive ionization mode due to different retention times. Indeed, the extracted chromatogram peak of two standards, *PC (14:0/14:0)* and *PE (14:0/14:0)*, which was evaluated in RPC and HILIC, illustrates this behavior (Fig. 6). Due to distinct retention times and nonoverlapping peaks, it was observed that the separation between these classes was more efficient in HILIC. Furthermore, when both chromatograms were compared, *PC (14:0/14:0)* eluted before *PE (14:0/14:0)*, in RPC, whereas *PC (14:0/14:0)* eluted after *PE (14:0/14:0)*, in HILIC, indicating that PC might be more polar than PE (Fig. 6). However, this assumption is made for two lipid species that differ only by their polar head. Other factors, such as the number of double bonds and carbon number interfere with this separation and thus the specific retention time of each species varies significantly within class. The same behavior was observed for other endogenous PE and PC species in the analyzed samples. Other lipid subclasses such as LPE could be detected in either positive or negative mode due to their ionization efficiency in both ionizations, whereas LPC were only detected in positive mode. We also demonstrated the LPC and LPE tend to coelute in RPC, however these classes are efficiently separated in HILIC, as we have shown through the behavior of the two IS (Fig. 6). Furthermore, we show that PS were also detected in positive and negative ionization modes. This lipid class presented isobaric species along with PC in negative mode. PS is detected as a deprotonated adduct in negative ionization mode, whereas PC was detected as an acetate adduct [M+OAc]^-^ in negative ionization mode. For instance, PS (36:0) and PC (32:1) are two isobaric species with an exact mass at *m/z* 790.5604 in negative mode. Their separation cannot be performed in RPC as these classes coelute (RT: 4.5–9 min). For the quantification of these species this can be an issue as these lipids cannot be separated through our method, therefore we chose to relatively quantify these species only in positive mode, in which they share distinct *m/z*. The negative ionization mode is however very beneficial for the annotation process. For instance, the analysis of the PC in the negative mode, as an [M+OAc]^-^ adduct, was the basis for the annotation process of these molecular species to confirm the nature of the FFA and lysoforms. Overall, the separation of these classes, particularly GPh, lyso-GPh, Cer, and derivatives such as HexCer, Hex2Cer, can be performed more efficiently through HILIC mode, where intraclass differentiation is far more sensitive as the separation is primarily based on their polarity. Nonetheless, RPC remains an effective method for within class separation. For example, within PE class, lipids can be distinguished based on the hydrophobicity which is favored by the presence of fatty acyl chains, which contributes to the molecule’s hydrophobicity and thus separates more efficiently these lipids. Classes such as Cer, HexCer, Hex2Cer, sphingosine bases, and SM also presented a better sensitivity in HILIC, implying that this method might be a better option when analyzing these species. Also, PE, LPE, PG, PI, and FA lipids were detected in negative in RPC and HILIC. Moreover, FA responded better in negative ionization mode but represented a far better response in HILIC in negative than in RPC.

## DISCUSSION

### RPC and HILIC are complementary methods for global lipid analysis

Accurate lipidomic studies in biological tissues/biofluids provide the basis for understanding molecular pathways of diseases associated with lipid metabolism dysregulations, such as AMD (28, 34, 35). Most of lipidomic research is focused on targeted techniques. Understanding how metabolic pathways may be involved in disease onset and progression requires a comprehensive representation of nearly all lipid classes. On the other hand, absolute quantification through untargeted techniques is challenging due to a lack of IS for many endogenous species. It is now acceptable to use one IS per class, although it may result in errors because molecules from the same class have distinct properties (36, 37). Likewise, lipids are very complex structures ranging from highly polar to hydrophobic. These characteristics make it difficult to design a global analytical procedure through a single analytical run and such studies have not yet been reported to our knowledge. We attempt to present the benefits of a dual analytical method of using RPC and HILIC, alternatively, to identify and quantify the relative amounts of about 15 lipid classes and approximately 500 lipid species, in positive and negative ionization modes, from four different biological tissues/biofluids (erythrocytes, retina, RPE/Choroid, plasma). Lipid classes/subclasses were annotated through the combination of the *LipidSearch* software and additional manual spectra elucidation, which warrants a robust and reliable annotation process. We highlight the benefits and drawbacks of the RPC and HILIC method, for analyzing lipid classes, as well as pointing out their sensitivity based on lipid separation and response in both ionization modes. Based on the biological matrices that were analyzed, such information can be relevant, as some lipids exhibit better response through RPC rather than HILIC. Although both chromatographic methods can separate a wide range of lipid classes, it is understandable that using one or the other alone has some drawbacks, as some lipid species have a better signal with one instead of the other. We suggest that the complementarity of these chromatographic methods is highly recommended in untargeted lipidomic approaches which can be adapted to analyze lipids in a global approach. The current developed combination technique has several advantages, including (i) short time analysis (10 min for HILIC and 17 min for RPC), which can be used for long series analysis, (ii) coupling of these chromatographic methods with HRMS which guarantees high resolution in terms of mass exactitude, and (iii) the applicability of these methods on almost all biological tissues and biofluids. Such an approach can be highly beneficial when analyzing lipids of a certain category in specific tissues. For instance, in ocular tissues, such as the human retina, GPh are major classes while other classes are less abundant. In this case, the HILIC method is preferable for lipid analysis. In contrast, for the analysis of human plasma, where Chol, CE, and TAG are abundant, the RPC may be the preferred method. We show that RPC approach in untargeted lipidomics tends to separate better species such as Chol, CE, DAG, and TAG. Other conditions in addition to chromatographic separations might interfere with this behavior, such as ionization sources, although they were not studied in this work. When an ESI source is used, for example, CEs tend to fragment in source. This fragmentation results in the formation of dehydrated cholesterol, reducing the detection and relative quantification of CE. Furthermore, it has been suggested that an atmospheric-pressure chemical ionization source may also be an appropriate method to analyze CE (33).

The advantages of HILIC are primarily focused on the separation of GPh. Such separation is also achieved in RPC but it is more challenging to achieve intraclass separation for GPh because these classes tend to coelute. Also, the presence of isobaric molecular species that are either in positive ionization (between PE and PC or LPE and LPC) or negative ionization (PS and PC) were not differentiated in RPC. However, due to different RT, which can distinguish these species, it was possible to separate them in HILIC.

Lipid annotation is the most challenging process in lipidomics. This is due in part to the large number of lipid species and the inability to detect and represent all lipid classes in a single analytical run. Furthermore, the annotation process has not yet been fully automated, even though several software, such as *LipidSearch*, *MZmine* (http://mzmine.github.io/), *MS-Dial* (http://prime.psc.riken.jp/compms/msdial/main.html) and others provide a good approach. In comparison to metabolomics software, lipidomics, and data processing software, particularly the annotation process, is still in development. Based on those developments, we performed lipid annotation using a combination of *LipidSearch* and manual annotation. We were able to investigate the spectra of all ions obtained in HILIC and RPC in positive and negative ionization modes in MS^2^. Chol, CE, DAG, and TAG, for example, were not detected in HILIC. As a result, in order to annotate these species, we investigated the parent ion of this species as ammonium adducts [M+NH_4_]^+^ using the RPC method. These methods' complementarity was critical for the overall identification and annotation of lipid species.

We evaluated the overall lipid distribution for RPE/Choroid and retina in RPC and HILIC in positive and negative ionization modes. We demonstrated that lipid distribution of certain lipid classes differed between subjects. For instance, in the retina, *subject 10* presented a higher HexCer than other subjects. No such result was observed for RPE/Choroid. Dysregulated metabolic process might explain these results. Prior studies have suggested that high retinal glucosylceramide content is present in individuals suffering from diabetic retinopathy. The overproduction might be due to the augmented uridine diphosphate glucose production through the pentose pathway, suggesting that hyperglycemia in diabetes induces glucosylceramide production (38). However, we hypothesize that potential dysregulated sphingolipid metabolism in retinal tissues could explain the abnormal lipid degradation that results in higher HexCer in the retina, pointing out to potential directions for further research into sphingolipids as potential targets in retinal degenerations. We do not exclude the necessity of targeted approaches directly analyzing specific lipids, in order to better understand these differences in lipid distributions. Furthermore, based on the comparison of two methods the relative distribution of lipid classes differs. That was mostly evidenced for the Hex2Cer, which presented a higher intensity in the RPC than HILIC. The separation of this class can be performed more efficiently through HILIC than RPC. In RPC, Hex2Cer coelutes with other species, including mostly phospholipid subclasses. In contrast, in HILIC, Hex2Cer elutes at a retention time that is easily distinguishable from that of phospholipids and other classes. Typically, we rely on the RT time for identification. We hypothesize that the coelution effect and matrix ionization processes may induce competition between ions, particularly between phospholipids (which occupy nearly 50 % of the retina) and Hex2Cer. This behaviour may hinder the detection of these species, as well as their relative intensity, which may contribute for the “overestimation” of this class in RPC. For the detection and relative quantification of Hex2Cer and other sphingolipid subclasses, such as (Cer and HexCer, we recommend the HILIC method, which provides a more accurate separation based on retention times that do not overlap, than RPC.

Higher lipid distribution was observed in RPE/Choroid for LPC in positive mode and FFA in negative mode, implying GPh lipid degradation due to prior sample manipulation or storage. Although all necessary precautions were taken to ensure proper sample manipulation, we hypothesize that previous sample withdrawal in hospital facilities should be considered in order to avoid degradation resulting from improper manipulation such as leaving samples on the bench for prolonged periods of time, repetitively performing analysis, and thawing. Such variations, while seemingly insignificant, can promote enzymatic activities that lead to lipid degradation. These variables might induce errors when performing lipid analysis in biological tissues and we acknowledge, therefore we recommend considering these factors during analysis to minimize them if possible. However, more in-depth investigation and targeted quantification is required, especially by targeting specific compounds to determine if disrupted metabolism or simply poor sampling manipulation interferes with lipid distribution, which might be performed between control and diseased subjects. These suggestions highlight the importance of evaluating lipids that share same metabolic pathways to illustrate more concretely these lipid disturbances. Overall, this methodological approach provides a comprehensive technique as well as an explanatory analysis of how different lipids respond to different analytical separation techniques, which needs to be considered for detailed and accurate lipid identification and quantification. In addition, these findings could be beneficial to the lipidomic community, particularly those focusing on lipid metabolism dysregulations especially those related to retinal degenerations such as AMD.

## Data availability

Data that support the plots within this publication and other findings of this study are available from the corresponding authors upon reasonable request.

## Supplemental data

This article contains supplementary data.

## Conflict of interest

The authors declare that they have no conflicts of interest with the contents of this article.
