# Supplementary material for: Comprehensive mass spectrometry lipidomics of human biofluids and ocular tissues
Source: J Lipid Res. 2023 Feb 10;64(3):100343. doi: 10.1016/j.jlr.2023.100343 (PMC10027555; doi:10.1016/j.jlr.2023.100343)
Supplement: Supplemental Table S5 [file mmc5.docx]

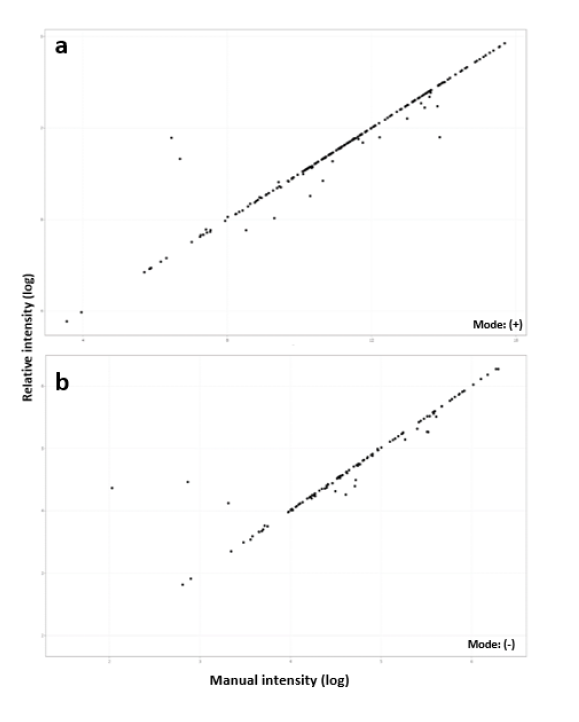


**Supplemental Figure S3 :** Representation of lipid’s intensity correlation between automatic processing through R and manual extraction of peak’s intensity (logarithmic scale) between relative and manual intensity of extracted molecular ions in positive mode (*correlation coefficient : 0.996*) **(a)** and negative mode (*correlation coefficient: 0.997*) **(b)**.
